# Prospective Randomized Multicenter Comparison of the Clinical Outcomes of V4c and V5 Implantable Collamer Lenses: A Contralateral Eye Study

**DOI:** 10.1155/2018/7623829

**Published:** 2018-09-05

**Authors:** Takashi Kojima, Yoshihiro Kitazawa, Tomoaki Nakamura, Masahide Takahashi, Kazutaka Kamiya, Kazuo Ichikawa, Akihito Igarashi, Kimiya Shimizu

**Affiliations:** ^1^Nagoya Eye Clinic, Nagoya, Japan; ^2^Kobe Kanagawa Eye Clinic, Tokyo, Japan; ^3^School of Allied Health Sciences, Kitasato University, Kanagawa, Japan; ^4^Chukyo Eye Clinic, Nagoya, Japan; ^5^Department of Ophthalmology, Sanno Hospital, Tokyo, Japan

## Abstract

**Purpose:**

To compare the visual and refractive outcomes and night vision performance questionnaire results between V4c and V5 implantable Collamer lenses in a prospective, randomized, multicenter study.

**Settings:**

Four refractive surgery centers.

**Design:**

Prospective randomized multicenter single-masked comparative study.

**Methods:**

Twenty-three patients were enrolled in this study. A conventional V4c model (EVO Visian ICL) was implanted in one eye, and a V5 model (EVO+ Visian ICL), which has a larger optic diameter than the V4c model, was implanted in the contralateral eye. The uncorrected distance visual acuity (UDVA) and corrected distance visual acuity (CDVA) were evaluated before and 6 months after surgery. At 6 months after surgery, a questionnaire on night vision disturbances was administered. The efficacy, safety, and predictability of the two implanted ICL models were compared.

**Results:**

There were no significant differences in the postoperative UDVA and CDVA between the two ICL models. The mean efficacy indexes for the V4c and V5 lenses were 1.16 ± 0.22 and 1.03 ± 0.23, respectively. The mean safety indexes of the V4c and V5 lenses were 1.21 ± 0.20 and 1.19 ± 0.20, respectively. The night vision performance questionnaire revealed that 7 patients (37%) noticed a difference in visual performance between the eyes, and all of them reported that they could see better at night with the V5-implanted eye compared with the V4c-implanted eye.

**Conclusion:**

The V4c and V5 ICL models achieved similar visual and refractive outcomes, whereas the V5 model showed a possible advantage in reducing night vision disturbances.

## 1. Introduction

The EVO Visian implantable Collamer lens (ICL, STAAR Surgical, Monrovia, CA, USA) is a posterior chamber phakic intraocular lens that has been widely used for refractive surgery. The ICL can be used regardless of corneal thickness and topography. Several studies have demonstrated its long-term efficacy and safety for patients with moderate to high myopia [1–3]. Recent studies also showed its efficacy and safety for low myopia and for the eyes with early keratoconus, and the ICL indication has expanded [4–8].

The V4 model implantable Collamer lens was introduced in 1998. It was designed to achieve a sufficient vault, which is the distance between the crystalline lens and the ICL. The V4c model, which has a central hole, was introduced in 2011. The V4c (EVO Visian ICL featuring KS-AquaPORT) model was designed to prevent secondary cataracts after surgery by providing aqueous flow through the central hole. In 2016, the V5 model (EVO+ Visian ICL), with a larger optical diameter, was introduced. The V5 model has an optical diameter of 5.0–6.1 mm depending on the lens power, while the range of the conventional V4c model is 4.9–5.8 mm.

In relation to overall patient satisfaction after ICL implantation, 88% of high myopic patients were satisfied or very satisfied according to the questionnaire, which used a Quality of Life Impact of Refractive Correction [9]. However, patients who were dissatisfied with the surgery reported dissatisfaction with night vision.

Night vision disturbances after corneal laser refractive surgery, including photorefractive keratectomy (PRK) or laser in situ keratomileusis (LASIK), have been reported as the primary factors affecting patient satisfaction [10–15]. Lim et al. reported that 26% of patients reported glare and 34% reported halos after ICL implantation [16]. They also reported that the frequency of halos increased when the difference between the optical diameter of ICL and the pupil size increased. These results suggested that light passing through the outside of the optical zone caused halo symptoms when the pupil diameter became larger than the optical zone of the ICL.

A previous study used optical simulation experiments to investigate the optical quality difference between the V4c and V5 ICL models. These experiments revealed no significant differences in higher order aberrations, the Strehl ratio, or the point spread function [17]. However, there are no reports comparing the clinical results of the two models.

In the current study, we implanted a V4c model in one eye and a V5 model in the other eye of patients, and we investigated the difference between visual/refractive outcomes and a patient questionnaire regarding visual disturbances at night.

## 2. Materials and Methods

The study was performed as a multicenter study and included Kitasato University, Nagoya Eye Clinic, Kobe Kanagawa Eye Clinic, and Sanno Hospital in Japan. Patients >19 years old who agreed to participate in the study were enrolled. Patients who had ocular diseases other than refractive errors, and those who had a history of ocular surgery including laser refractive surgery, were excluded.

The V4c model was implanted in one eye, and the V5 model was implanted in the other eye. The ICL model was randomly assigned using the envelope method and was masked from the patients. Patients who asked us to inform them of the ICL model in each eye were informed after the 6-month follow-up examination.

All ICL implantation surgeries were performed using a standardized method in all surgical centers. Briefly, a 1.0-mm side-port incision was created, and the anterior chamber was filled with viscoelastic material. Then, a 3.0-mm temporal clear corneal incision was created, and the ICL was inserted into the anterior chamber, resting on the iris. The four haptics of the ICL were inserted behind the iris with a specially designed manipulator. Then, irrigation and aspiration were performed, and the pupil was constricted with an intracameral acetylcholine injection.

The size of the ICL was selected based on the nomogram provided by STAAR Surgical. Manifest refraction, uncorrected distance visual acuity (UDVA), and corrected distance visual acuity (CDVA) were measured before and after surgery. The vault was measured using a slit-lamp based on previous reports [18]. A moderate vault was defined as more than 0.5 times and less than 1.5 times the central corneal thickness (CCT). A vault more than 1.5 times the CCT or less than 0.5 times the CCT was defined as a high or a low vault, respectively.

At 6 months after surgery, the efficacy index (postoperative UDVA divided by preoperative CDVA) and safety index (postoperative CDVA divided by preoperative CDVA) were calculated.

This study was approved by the Institutional Review Board and adhered to the tenets of the Declaration of Helsinki, and the study protocol was uploaded in the clinical trial registration site (UMIN000032396). Informed written consent was obtained from all patients after explanation of the nature and possible outcomes of the study.

### 2.1. Night Vision Questionnaire

A questionnaire regarding night vision disturbances was administered 3 months after surgery. After explaining halos and glare symptoms, we asked patients to answer the questions. Glare was explained as difficulty seeing objects due to scattered bright light, and halo was explained as a bright circle surrounding a light source, according to previous reports [13, 16].

### 2.2. Statistical Analyses

A Wilcoxon matched-pairs signed-rank test was performed to compare the parameters between the two groups. A Chi-square test was performed to analyze the distribution of vault and refractive error between the two groups. A *p* value < 0.05 was considered significant.

## 3. Results

### 3.1. Patient Backgrounds

A total of 23 patients were enrolled in this study. Among them, 19 patients completed examinations 6 months after surgery. Patient preoperative demographic information is shown in Table 1. There were no significant differences in visual function, manifest refractive power, or biometry between the eyes.

### 3.2. Efficacy

All patients in both groups had UDVA ≥20/30. In total, 100% (19 eyes) of the V4c group and 89.5% (17 eyes) of the V5 group showed UDVA ≥20/20, respectively. There were no significant differences in UDVA between the V4c (20/12, −0.23 ± 0.09 logMAR) and the V5 groups (20/13, −0.19 ± 0.12 logMAR) (*p*=0.152) 6 months after surgery. The efficacy index for the V5 group (1.03 ± 0.23) at 6 months was statistically significantly different from that of the V4c group (1.16 ± 0.22; *p*=0.044) (Table 2).

### 3.3. Safety

There were no significant differences in the 6-month postoperative CDVA between the V4c (20/12, −0.25 ± 0.07 logMAR) and V5 groups (20/11, −0.26 ± 0.07 logMAR; *p*=0.50). The safety index of the V5 group at 6 months (1.19 ± 0.20) was not significantly different from that of the V4c group (1.21 ± 0.20; *p* > 0.999; Table 2). None of the patients in either group lost two or more lines of CDVA.

### 3.4. Predictability

A total of 94.7% (18 eyes) and 84.2% (16 eyes) were corrected to within ±0.5 D of the target refraction in the V4c and V5 groups, respectively. All eyes in both groups were corrected to within ±1.0 D of the target refraction. There was no significant difference in distribution corrected to within ±0.5 D of the target refraction between the two groups (*p*=0.29).

### 3.5. Vault

In the V4c group, 1, 14, and 4 eyes showed a low, moderate, or high vault, respectively, at 6 months after surgery. In the V5 group, 15 and 4 eyes showed a moderate and high vault, respectively. There were no significant differences in vault distribution between the two groups (*p*=0.596).

### 3.6. Patient Answers to the Questionnaire

**Figure 1:**
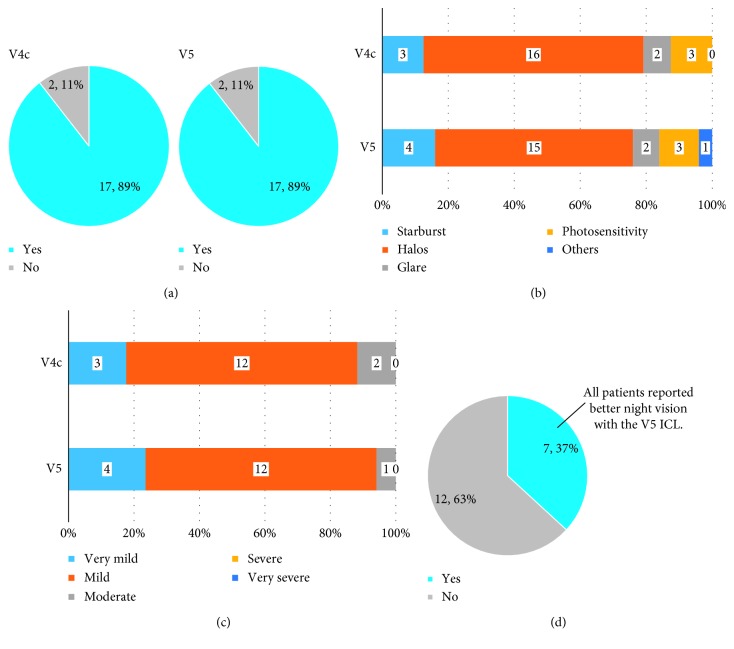
Results of the questionnaire concerning visual performance at night. Four questions regarding night vision disturbances were asked as follows. (a) Did you notice changes in your night vision after surgery? (b) What changes in night vision did you notice? (Multiple answers are allowed.) (c) How severe are your symptoms? (d) Did you notice any difference between eyes when looking at a light source in a dark place? If yes, please tell us which eye sees better at night.

In both groups, 89% (17 eyes) of the patients reported a change in night vision (Figure 1(a)). For patients who noticed changes, we asked about the changes in detail. Sixteen patients in the V4c group and 15 in the V5 group reported halos (Figure 1(b)). However, none reported severe or very severe symptoms (Figure 1(c)). When we asked about differences between the eyes when seeing a light source at night, seven (37%) patients reported there were some differences. All patients who reported differences between the eyes declared that they could see better at night with the eye that received the V5 ICL model (Figure 1(d)). Moreover, we asked these patients to demonstrate the difference by drawing it. All patients reported that the extent of the halo was less in the V5 eye compared with the V4c eye (data not shown).

### 3.7. Complications

One patient showed an extreme high vault of approximately 3 times the corneal thickness in both the eyes. At 3 months after surgery, the surgeon rotated each ICL 90°, and fixed them in a perpendicular position. Repositioning surgery was successfully performed in both the eyes, and the vaults decreased immediately after surgery. At 6 months after surgery, the vaults were 1.5 times the corneal thickness in both the eyes.

## 4. Discussion

The current study compared the efficacy, safety, and predictability of the V4c and the V5 ICL models. Both groups showed high efficacy, safety, and predictability, which were similar to previous studies [8, 19]. However, the average efficacy index in the V4c group was significantly higher than that in the V5 group. We found that two eyes with V5 ICL implantation showed UCVA lower than 20/20 due to the undercorrection of myopia and astigmatism. Since the average efficacy indices in both groups were greater than 1.0, we believe that surgery using both ICL models may be considered a highly effective refractive surgery. However, further studies including a large number of cases are warranted to clarify the difference of efficacy between the two models.

In both groups, 79% of eyes showed a moderate vault and 21% showed a low vault. From these findings, we concluded that we can select the ICL size with the same method used for the V4c model. However, as the peripheral part of the V5 model is thicker than the V4c model, the peripheral part of the V5 model may be close to the crystalline lens in cases of low vault. Further studies are needed to investigate the peripheral vault in the V5 model.

The patient questionnaire revealed that 89% of patients noticed changes in light perception, which they did not experience before surgery, and that most of these symptoms were halos. However, none reported severe or very severe symptoms. Overall severity of night vision disturbances was not severe compared with previous studies evaluating PRK and LASIK postsurgical outcomes [13, 20, 21]. Better optical quality of the eyes after ICL implantation than that after corneal laser refractive surgery may be the reason [4, 15].

Moreover, we asked patients about the differences in light perception between the eyes. Surprisingly, 37% of patients reported a difference, and all reported that they could see better using the eye with the V5 model. We asked these patients to draw the difference, and found that halos were milder in the eye with the V5 model compared to the eye with the V4c model. To evaluate the factors affecting night vision disturbances, we compared age, gender, and ICL size between those patients with and those without a difference in light perception at night between both the eyes (data not shown). However, we could not find any significant parameters. Future studies which include a large number of cases may reveal who can benefit from the V5 model.

Koch et al. reported the pupil size in various situations, including driving and reading at night [22]. Apparent pupil size at the corneal plane is 1.26 times larger than the actual size when the *K* value is 40 D and the anterior chamber depth is 3.5 mm [23]. Based on this equation, the apparent optical zone of the V5 model is estimated to be 6.3–7.6 mm (actual size; 5.0–6.1 mm). However, the apparent pupil size in the V4c eyes was estimated to be 6.2–7.3 mm (actual size; 4.9–5.8 mm). We speculated that these differences in the optical zone may have caused the different halo symptoms between the eyes with V4c and V5 ICLs.

One patient showed a very high vault in both the eyes after surgery. Since a previous report has shown that sulcus to sulcus diameter in the vertical position is greater than the horizontal diameter [24], surgical rotation of the ICL was performed. The very high vault decreased immediately after surgery. There were no intraoperative or postoperative complications such as pigment dispersion syndrome and cataract formation. Although toric ICLs cannot be rotated, treatment by rotation for cases with extreme high vault could be an option for nontoric ICLs. In this study, 21% of patients showed a low vault. Further studies are needed to avoid inappropriate vaults.

There are several limitations in the current study. Although the selection of ICL models was masked for patients, it was not masked for examiners. Bias by examiners could have been introduced during the measurement of visual and refractive outcomes. Moreover, we adopted a contralateral eye study. Since an improved model was used for one eye, a study including a large number of cases was not accepted by the Institutional Review Board. For this reason, this study included a small number of cases. A large case-control study comparing different patients with V4c and V5 models may be necessary. In the current study, we could not analyze the pupil size due to different conditions at the participating surgery centers, including differences in luminance levels in the examination rooms and the use of different examination instruments. As a larger mesopic pupil size compared with the ICL optical zone is one risk of night vision disturbance, [16] future studies should investigate if it is possible to assess the risk of night vision disturbances after ICL surgery by measuring the pupil size. We compared clinical outcomes between the two groups 6 months after surgery. Although the clinical outcomes including visual acuity and refractive power became stable 1 month after ICL implantation surgery, [25] long-term follow-up is necessary in future studies.

In conclusion, the V5 ICL model presented similar efficacy, safety, and predictability compared with the conventional V4c model and showed a possible advantage in reducing night vision disturbances.

## Figures and Tables

**Table 1:** Preoperative patient demographic information.

Variable	V4c	V5	*p* value
Age (years)	32.4 ± 5.7		—
Gender	Male 9; Female 10		—
UDVA (logMAR) (Snellen)	1.30 ± 0.26 20/400	1.31 ± 0.22 20/400	0.814
CDVA (logMAR) (Snellen)	−0.17 ± 0.09 20/14	−0.19 ± 0.07 20/13	0.250
Manifest sphere (D)	−7.14 ± 3.46	−6.96 ± 2.78	0.572
Manifest cylinder (D)	−0.34 ± 0.40	−0.26 ± 0.32	0.574
Spherical equivalent (D)	−7.32 ± 3.42	−7.09 ± 2.84	0.278
ACD (mm)	3.04 ± 0.29	3.04 ± 0.30	0.471
WTW (mm)	11.55 ± 0.45	11.53 ± 0.40	0.804

UDVA: uncorrected visual acuity; CDVA: corrected distance visual acuity; ACD: anterior chamber depth; WTW: white to white (horizontal corneal diameter). Values are presented as mean ± standard deviation.

**Table 2:** Comparison of visual and refractive outcomes between the V4c and V5 models 6 months after surgery.

Variable	V4c	V5	*p* value
UCDVA (logMAR) (Snellen)	−0.23 ± 0.09 20/12	−0.19 ± 0.12 20/13	0.152
CDVA(logMAR) (Snellen)	−0.25 ± 0.07 20/11	−0.26 ± 0.07 20/11	0.500
Manifest sphere (D)	0.20 ± 0.30	−0.22 ± 0.42	0.497
Manifest cylinder (D)	−0.29 ± 0.29	−0.27 ± 0.22	0.125
Spherical equivalent (D)	0.05 ± 0.07	−0.09 ± 0.38	0.505
Efficacy index	1.16 ± 0.22	1.03 ± 0.23	0.044
Safety index	1.21 ± 0.20	1.19 ± 0.20	>0.999

UCDVA: uncorrected distance visual acuity; CDVA: corrected distance visual acuity. Values are presented as mean ± standard deviation.

## Data Availability

The data used to support the findings of this study are available from the corresponding author upon request.
